# Addressing Structural Inequities, a Necessary Step Toward Ensuring Equitable Access to Telehealth for Medication Abortion Care During and Post COVID-19

**DOI:** 10.3389/fgwh.2022.805767

**Published:** 2022-03-17

**Authors:** Terri-Ann Thompson, Dana Northcraft, Fabiola Carrión

**Affiliations:** ^1^Ibis Reproductive Health, Cambridge, MA, United States; ^2^Expanding Medication Abortion Access (EMAA) Project, Washington, DC, United States; ^3^National Health Law Program, Los Angeles, CA, United States

**Keywords:** telehealth, telemedicine, medication abortion, equity, racial and ethnic disparities, reproductive justice, Medicaid, COVID-19

## Abstract

Telehealth, one of the newest health innovations, has been promoted as a tool to enhance access to health care services in ways that center patient needs. However, integrating telehealth within an inequitable health system undermines its potential. This perspective highlights policies and practices that foster structural inequities and names their impact on the use and acceptability of telehealth for medication abortion among specific communities of color. Communities of color have a higher prevalence of abortion use but face many barriers, including financial and geographic barriers, to abortion access. Preliminary evidence on telehealth for medication abortion shows that it is highly acceptable, accommodating of patient needs, and may allow patients to access abortion care at earlier gestational ages. However, evidence during the COVID-19 pandemic shows that utilization of telehealth is lower among communities of color. We describe how systemic barriers, including regulations on or laws banning telehealth for medication abortion, disinvestments in digital access, and restrictions on public insurance coverage, could perpetuate lower utilization of telehealth for medication abortion care among communities of color. We call for systems changes that will remove these barriers and make this health care innovation available to all who may desire it.

Every person should have the right to “decide the number, spacing, and timing of their children, to have the information and means to do so, and to attain the highest standard of sexual and reproductive health” (1). However, for many communities of color in the United States (US), this right goes unrealized. Sociodemographic factors such as socioeconomic status, residence, race, and ethnicity, exclusively and mutually, shapes their reproductive health experience and has resulted in calls for the use of a reproductive justice framework (2); one that acknowledges the role of societal factors in the achievement of good sexual and reproductive health.

Research indicates that health disparities based on social status and race/ethnicity exist and persist in the US. In the abortion space, Black, Latinx, and low-income individuals have higher rates of abortion (3) due to higher rates of unintended pregnancy, a consequence of reduced access to and effective use of contraceptives (4). Simultaneously, these populations experience reduced access to abortion services (5). Racial and ethnic differences in abortion access can be tied to a higher proportion of Black and Latinx communities being publicly insured and/or without insurance coverage (6, 7), a higher proportion being low-income (8), and fewer abortion facilities being located in neighborhoods where more than half of the residents are Black or Latinx (9). The results of reduced abortion access are dire, with minority groups having disproportionately higher experiences of negative health and wellbeing outcomes such as increased financial insecurity (10), reduced aspirational life plans (11), and increased incidence of serious pregnancy complications such as eclampsia as well as poorer physical health for years post pregnancy (12).

One strategy for reducing abortion disparities is Telehealth^1^; the use of electronic information and telecommunications technology to facilitate health care. Telehealth has been used to provide assessment, counseling, and follow-up care for medication abortion (*a safe and effective abortion method where two medications, mifepristone and misoprostol, are taken to terminate a pregnancy)*. It has similar rates of effectiveness and safety as in-person provision of medication abortion care and is highly acceptable to patients (14). Additionally, telehealth models for medication abortion have been shown to be person-centered models; accommodating childcare and jobs, eliminating the need to travel for care, reducing abortion related costs such as accommodation, lost wages, and childcare, and maintaining privacy (15–17). Telehealth delivery of abortion care was in use prior to the COVID-19 pandemic, but its use surged during the pandemic to sustain access to services while observing restrictions on travel and in-person care (18, 19).

While telehealth has the potential to address barriers to abortion care and expand access, its full potential cannot be realized until policies and practices that foster structural inequities in healthcare generally, and specifically in abortion care and telehealth, are addressed. In this perspective we highlight a few of these policies and practices and how they limit the utilization and benefits of telehealth for medication abortion among Black and Latinx populations.

## Structural Inequities that Impact the Utilization and Acceptability of Telehealth for Medication Abortion

Data on the utilization of telehealth for reproductive services is limited. Pre-pandemic, analyses of private claims data found that telehealth accounted for <0.05% of reproductive health claims (contraceptive management, medication abortion, prenatal care, and STI testing and treatment) (20). Among the reasons for this low utilization is a preference for in-person care, low adoption of services by providers, and a belief that telehealth services would be less personal or of a lower quality of care (20, 21). Further, studies outside of abortion have highlighted disparities in telehealth use, with lower utilization seen among adults who were older, Black, self-paying, who had Medicaid, or Medicare status, and were from urban areas (22, 23). These disparities are particularly concerning because they occur among those who already face systemic barriers to health care. Addressing these disparities requires an explicit look at policies and practices that impact the utilization, adoption, and acceptability of telehealth for medication abortion and have discriminatory effects.

### Provision of Abortion Care

Abortion was a common medical procedure prior to the civil war, with a large number of practitioners being Black and Indigenous women (24). With the end of slavery came a cry for more White births, and an assertion for White men to control the medical field (25). The American Medical Association refused to admit women and Black people to its ranks and instead undertook a smear campaign to undo the status of midwives in American society (25). Abortion was criminalized, leaving only the privileged with access to safe abortion care (24). Although the constitutional right to abortion was established in 1973, there have been repeated attempts to limit practice and scope for both surgical and medication abortion. With regard to medication abortion, there are state restrictions on in-person (and thus, telehealth) dispensing of medication abortion medicines as well as on who can dispense abortion medications (26). These restrictions work to reduce the ability of Black and Latinx patients to enjoy the benefits of telehealth for medication abortion such as accommodation as well as to engage with telehealth for medication abortion with their provider of choice. These laws also perpetuate the multiple intersecting inequities these communities face that reinforce distrust of the medical system.

For more than twenty years, the Food and Drug Administration (FDA) has imposed significant restrictions on the dispensation and distribution of mifepristone, classifying it under a Risk Evaluation and Mitigation Strategy (REMS), allowing for the imposition of restrictions usually only placed on a limited number of drugs. The three REMS requirements were: (1) mifepristone must be dispensed only in a clinic, hospital, or under the direct supervision of a certified medical provider; (2) the prescribing provider must be certified in the mifepristone REMS Program by submitting a Prescriber Agreement Form to the drug distributor; and (3) the patient must sign and the provider must obtain the FDA-approved Patient Agreement Form (27). As a result of the efforts of reproductive health, rights, and justice advocates, on December 16, 2021 the FDA announced it will remove the in-person-dispensing requirement of mifepristone, permanently allowing its dispensing through mail or delivery. (28) While the FDA made it possible for any type of pharmacy to dispense mifepristone, it added a new requirement that these pharmacies be certified.

Before the COVID-19 pandemic, telehealth for medication abortion care was available in a site-to-site, or clinic-to-clinic, manner. In this model, the patient had to travel to a clinic to be physically handed the pills due to the REMS (26). Depending on geographic location, this may have required some patients to travel hours or hundreds of miles to receive pills dispensed after a telehealth visit (clinical evaluation, counseling, consenting). Only patients enrolled in an FDA approved clinical trial could receive direct-to-patient care and be sent medications by mail. Modeling done during the pandemic showed that nearly 2 million clinical abortion contacts could have been avoided if the REMS had been removed (29). Given that 62% of abortions are provided to Black, Latinx, and other people of color (30), the risk of exposure to COVID-19 was disproportionately higher for this population seeking this essential reproductive service than their White counterparts.

Thirty-three (33) states only allow physicians to dispense mifepristone. These laws prevent non-physicians such as nurse-midwives, nurse practitioners, and physician assistants, from providing medication abortion (26). This restriction is not only harmful because of the growing role of advanced practice clinicians (APCs) in providing clinical services to underserved populations, (31) but also because preliminary evidence shows that nurse-centered care can alleviate medical mistrust (32), due to a history of medical racism (33). Restricting who can dispense the medications disproportionately disrupts continuity of care for non-White abortion seekers.

### Service Delivery

Despite evidence of its safety and effectiveness, 19 states currently either ban telehealth for medication abortion care or require in-person visits, which effectively bans the use of telehealth (26). Eleven of these states are considered part of the south; states where Black and Latinx communities are highly represented (34), that limited access to abortion facilities and providers (35), and that amplified abortion restrictions during the pandemic (36). Without telehealth for medication abortion, Black and Latinx patients in these states may have to travel long distances to access abortion care. Research shows that Black, immigrants, and other people of color are less able to travel long distances to obtain abortion care because of reduced material and social resources (37).

During the COVID-19 public health emergency, the federal government took various initiatives to encourage the utilization of telehealth to allow for social distancing and reduce unnecessary clinical contacts (38). Additionally, the Centers for Medicare & Medicaid Services (CMS) published telehealth guidance to facilitate widespread adoption of telehealth services in state Medicaid programs (39). However, the Trump Administration denied the American College of Obstetricians and Gynecologists and reproductive justice organizations' requests to lift the in-person dispensing of mifepristone, that would have allowed for the broader use of telehealth delivery of abortions (40). Given findings that Black and Hispanic women were more worried about being able to afford or obtain contraceptive services than their White counterparts during the COVID-19 pandemic (41), this denial placed this population at further disadvantage.

### Insurance Coverage

Since 1976, an annual appropriations bill rider known as the Hyde Amendment has restricted federal funding for abortion services in Medicaid and other federal programs. The Hyde Amendment was designed to stop people living in poverty from having abortions, creating a de facto ban that would strip people of their constitutionally protected reproductive rights. Representative Henry Hyde, the sponsor who first introduced and long championed the Amendment, laid bare his intent: “I would certainly like to prevent, if I could legally, anybody having an abortion, a rich woman, a middle class woman, or a poor woman. Unfortunately, the only vehicle available is the… Medicaid bill (42).”

In its most recent version, the Hyde Amendment only requires abortion coverage in the narrow circumstances of rape, incest, or life endangerment (43). Thirty-three states cover abortions only as permitted by the Hyde Amendment and one (South Dakota) only covers abortions under life endangerment, violating federal Medicaid law (44). The impact of the Hyde Amendment has reverberated in state coverage laws where 26 states restrict abortion coverage in plans offered through health insurance exchanges and 11 states prohibit abortion coverage in private plans (44). Coverage restrictions limit access to abortion in all delivery formats; effectively placing telehealth for medication abortion out of reach for Medicaid beneficiaries, where Black and Latinx communities are vastly overrepresented because of income inequality and other economic challenges faced by this group.

### The Digital Divide

A past filled with disinvestments and isolation of communities of color keep telehealth from reaching its full potential. Initially, this can be tied to the practice of redlining, a now outlawed practice of manipulating home lending and ownership that is strongly associated with segregation in neighborhoods, separating Black from Whites. This separation was associated with increasing investments in White neighborhoods, such as health centers, grocery stores, and green spaces, and few to no such investments in Black neighborhoods (45). While officially outlawed in 1968, with the Fair Housing Act (46), the impact of redlining is still found today in the increased health disparities and more recently, stronger impacts of COVID-19 in Black and Latinx neighborhoods (47).

The impact can also be traced to a new form of redlining in digital form, found when neighborhoods populated predominately by people of color lack access to the internet and other technology resources compared to wealthier, White neighborhoods (48). While there are many advancements in broadband access on the horizon, mainstream discussion is focused on rural populations, which tend to be predominantly White (49). And even if broadband is available, it is not affordable for those living in historically oppressed and ignored areas, leaving them to rely on mobile-only communication options without smart phone capabilities (49). The result is reduced abilities for Black and Latinx communities to connect to telehealth. A comparison of telehealth engagement and modality use (video vs. telephone) pre- and post-pandemic in a mid-size city's academic medical system, found that among telehealth users, Non-English speakers, Black patients, Medicaid, and Medicare-insured were less likely to use video than telephone (50).

### Language Access

Language access is critical to navigating healthcare systems and the complex abortion care landscape. Language access can include oral interpreting, written translations, and provision of services directly in a non-English language (51). Various federal laws like the Title VI of the Civil Rights Act of 1964 and Section 1557 of the Affordable Care Act require language access services; states also have codified their own protections (52). However, there is a significant shortage of bilingual providers, translated documents, and medical interpreters in the healthcare system, as shown during the COVID-19 pandemic (53, 54). Language concordant care is further limited by reimbursement, with few insurers directly reimbursing for interpreter services and others reimbursing at a lower rate (55). In this context, adults with limited English language proficiency face language related barriers to care.

Initial studies during the COVID-19 pandemic demonstrate varied levels of acceptance of telehealth, with Black respondents favoring telehealth visits over Spanish-speaking Latinx respondents, in large part because of unaddressed language access issues (56).

## Actions Needed to Promote Equitable Access to Telehealth for Medication Abortion

Given the potential of telehealth to address inequities in health care access as well as its ability to offer care in ways that are patient centered, it is imperative policy and practice barriers be removed to ensure all the benefits of telehealth can be realized. In keeping with the reproductive justice framework, we believe systems changes will promote better reproductive autonomy and create communities in which people can thrive.

### Removing Barriers to the Provision of Abortion Care

The announcement that the FDA will permanently remove the in-person dispensing requirement for mifepristone solidifies telehealth as one service delivery method of medication abortion care. Results from a study out of the United Kingdom that examined what happens when in-person dispensing requirements are lifted, found that clinical outcomes for those using telehealth were equivalent to in-person care, abortion wait times were significantly reduced, and patients were able to access medication abortion care earlier in pregnancy (57). Similarly, new data from Gynuity's TelAbortion study from the US confirmed that providing medication abortion through telehealth and mailed medication is safe and effective (58). Among the nearly 1,400 abortions provided via telehealth, 95% were completed without a subsequent surgical procedure and 99% experienced no serious adverse events.

While the FDA's suspension of the in-person distribution requirement for mifepristone is a step in the right direction, more can be done. Patient and provider certifications, that provide no additional safety mechanisms, should be removed, as should the recently added pharmacy certification requirement.

Expanding the reach and acceptability of telehealth, will also require the removal of bans on telehealth delivery and restrictions on who can dispense the medications. Once telehealth for medication abortion is widely available, efforts must be made to facilitate integration of the service across multiple health facilities including community health centers, hospitals, in addition to abortion clinics. This will involve (1) instituting Federal grant programs that support the inclusion of telehealth and mobile integrated health to boost access to abortion care, and (2) a greater investment in resources for adults with limited English proficiency including reimbursement for medical interpreters by all insurers and adequate reimbursement rates.

### Removing Barriers to Health Care Coverage

To ensure all people have the same ability to choose from all abortion delivery options available, efforts must be made to enhance the abilities of reproductive health facilities to offer abortion and telehealth for medication abortion care. This requires first that abortion coverage be expanded across the United States and second that public and private insurance agencies as well as managed care plans recognize and reimburse for abortion care across different modalities (such as telehealth) and a range of providers. As such, Congress must end the Hyde Amendment and pass the Equal Access to Abortion Coverage in Health Insurance Act, which would restore insurance coverage of abortions in health programs and plans such as Medicaid (59) and make available all delivery models of abortion.

### Removing Barriers to Digital Access

Federal and state laws must be updated, and resources must be allocated for increased broadband connectivity and utilization of devices. A recent Congressional allocation of *$*65 billion in broadband investments is a welcome first step to improve telehealth access (60). Additionally, policymakers should allow consumers to use and permit Medicaid coverage of audio-only phones to access health services, like medication abortion. Approximately 97% of US residents own a cellphone of some kind (61), making this device a ready point of connection between provider and patient. Further, services administered in-person or via live-video conferencing should be paid at the same rate as audio-only services. Such coverage would particularly benefit Black and Latinx communities since they have the least access to broadband, computers, tablets, and smart phones (62).

## Conclusion

While telehealth is not the only solution to expand access to medication abortion, it should be considered part of a long-term, complementary, safe, and sustained strategy to improve access and convenience for every individual. During the COVID-19 pandemic, there has been rapid expansion in telehealth usage for various health services, including abortion care. It remains unclear whether telehealth addressed or exacerbated existing health inequities. However, now is the time to reflect on the system within which telehealth is being implemented and utilized, to work with communities of color, center a reproductive justice framework in the delivery of telehealth, and ensure systems are built that consider those facing the most hardships. Future research efforts should prioritize approaches that engage Black and Latinx communities in their formulation and implementation and seek to understand reasons for lower utilization of telehealth for medication abortion and the strategies that might address these challenges.

## Data Availability Statement

The original contributions presented in the study are included in the article/supplementary material, further inquiries can be directed to the corresponding author/s.

## Author Contributions

T-AT, DN, and FC are responsible for the conceptualization, data curation, and original draft preparation of the perspective. All authors contributed to the article and approved the submitted version.

## Funding

The development of this perspective received no external funding. However, the article processing charges were funded by the Barrus Medical Foundation, the Collaborative for Gender and Reproductive Equity, and an anonymous funder.

## Conflict of Interest

The authors declare that the research was conducted in the absence of any commercial or financial relationships that could be construed as a potential conflict of interest.

## Publisher's Note

All claims expressed in this article are solely those of the authors and do not necessarily represent those of their affiliated organizations, or those of the publisher, the editors and the reviewers. Any product that may be evaluated in this article, or claim that may be made by its manufacturer, is not guaranteed or endorsed by the publisher.

## Footnotes

^1^Terms used when describing telehealth: site-to-site (provider and patient at different facilities), direct-to-patient (provider at a facility and patient at another location, such as home), synchronous care (care that takes place in real time), asynchronous care (care that takes place through communication that occur at different times) (13).

## References

[1] United Nations Population Division | Department of Economic and Social Affairs. Available online at: https://www.un.org/en/development/desa/population/theme/rights/index.asp (accessed September 19, 2021).

[2] Ross LJ Solinger R. A reproductive justice history. In: Reproductive Justice [Internet]. 1st ed. Oakland, CA: University of California Press (2017). p. 9–57. Available online at: http://www.jstor.org/stable/10.1525/j.ctv1wxsth.4

[3] Jones RK Jerman J. Population group abortion rates and lifetime incidence of abortion: United States, 2008–2014. Am J Public Health. (2017) 107:1904–9. 10.2105/AJPH.2017.304042 29048970 PMC5678377

[4] Troutman M Rafique S Plowden TC. Are higher unintended pregnancy rates among minorities a result of disparate access to contraception? Cont Rep Med. (2020) 5:16. 10.1186/s40834-020-00118-5 33014415 PMC7527248

[5] Boonstra HD. Abortion in the Lives of Women Struggling Financially: Why Insurance Coverage Matters. Available online at: https://www.guttmacher.org/gpr/2016/07/abortion-lives-women-struggling-financially-why-insurance-coverage-matters (accessed September 9, 2021).

[6] Artiga S Hill L Orgera K Damico A. Health Coverage by Race Ethnicity, 2010-2019 [Internet]. San Francisco, CA: Kaiser Family Foundation (2021). Available online at: https://www.kff.org/racial-equity-and-health-policy/issue-brief/health-coverage-by-race-and-ethnicity/

[7] Kaiser Family Foundation. Medicaid Coverage Rates for the Nonelderly by Race/Ethnicity [Internet]. State Health Facts (2020). Available online at: https://www.kff.org/medicaid/state-indicator/nonelderly-medicaid-rate-by-raceethnicity/

[8] Wilson V. Racial Disparities in Income Poverty Remain Largely Unchanged Amid Strong Income Growth in 2019 [Internet]. Economic Policy Institute (2020). Available online at: https://www.epi.org/blog/racial-disparities-in-income-and-poverty-remain-largely-unchanged-amid-strong-income-growth-in-2019/

[9] Guttmacher Institute Claim That Most Abortion Clinics Are Located in Black or Hispanic Neighborhoods Is False. Available online at: https://www.guttmacher.org/claim-most-abortion-clinics-are-located-black-or-hispanic-neighborhoods-false (accessed September 8, 2021).

[10] Foster DG Biggs MA Ralph L Gerdts C Roberts S Glymour MM. Socioeconomic outcomes of women who receive and women who are denied wanted abortions in the United States. Am J Public Health. (2018) 108:407–13. 10.2105/AJPH.2017.304247 29345993 PMC5803812

[11] McCarthy MA Upadhyay U Ralph L Biggs MA Foster DG. The effect of receiving versus being denied an abortion on making and achieving aspirational 5-year life plans. BMJ Sex Reprod Health. (2020) 46:177–83. 10.1136/bmjsrh-2019-200456 32098771

[12] Gerdts C Dobkin L Foster DG Schwarz EB. Side effects, physical health consequences, and mortality associated with abortion and birth after an unwanted pregnancy. Womens Health Issues. (2016) 26:55–9. 10.1016/j.whi.2015.10.001 26576470

[13] Weigel G Ramaswarmy A Sobel L Salganicoff A Cubanski J Freed M. Opportunities Barriers for Telemedicine in the U.S. During the COVID-19 Emergency Beyond [Internet]. San Francisco, CA: Kaiser Family Foundation (2020). Available online at: https://www.kff.org/womens-health-policy/issue-brief/opportunities-and-barriers-for-telemedicine-in-the-u-s-during-the-covid-19-emergency-and-beyond/

[14] Endler M Lavelanet A Cleeve A Ganatra B Gomperts R Gemzell-Danielsson K. Telemedicine for medical abortion: a systematic review. BJO. (2019) 126 G:1094–102. 10.1111/1471-0528.15684 30869829 PMC7496179

[15] Fix L Seymour JW Sandhu MV Melville C Mazza D Thompson T-A. At-home telemedicine for medical abortion in australia: a qualitative study of patient experiences and recommendations. BMJ Sex Reprod Health. (2020) 46:172–6. 10.1136/bmjsrh-2020-200612 32665231

[16] Kerestes C Delafield R Elia J Chong E Kaneshiro B Soon R. “It was close enough, but it wasn't close enough”: a qualitative exploration of the impact of direct-to-patient telemedicine abortion on access to abortion care. Contraception. (2021) *104*, 67–72. 10.1016/j.contraception.2021.04.028 33933421

[17] Raymond E Chong E Winikoff B Platais I Mary M Lotarevich T. TelAbortion: evaluation of a direct to patient telemedicine abortion service in the United States. Contraception. (2019) 100:173–7. 10.1016/j.contraception.2019.05.013 31170384

[18] Koonin LM. Trends in the use of telehealth during the emergence of the COVID-19 pandemic–United States, January-March 2020. MMWR Morb Mortal Wkly Rep [Internet]. (2020) 69:1595–99. Available online at: https://www.cdc.gov/mmwr/volumes/69/wr/mm6943a3.htm 33119561 10.15585/mmwr.mm6943a3 PMC7641006

[19] The American Telemedicine Association. The Adoption of Telehealth [Internet]. (2021). Available from: https://www.americantelemed.org/wp-content/uploads/2021/05/Adoption-of-Telehealth.pdf (accessed September 9, 2021).

[20] Weigel G Frederiksen B Ranji U. Telemedicine in sexual and reproductive health. KFF. (2019).

[21] Robinson A Mitchell N Morgan I. Disconnects and digital divides: black women and birthing people's maternal telehealth experiences during the COVID-19 pandemic [Internet]. In: Black Maternal Health Virtual Conference. (2021). Available online at: https://www.nationalacademies.org/event/06-07-2021/docs/D1E4956CA4B84E431D1E181E1520D8EB44EC68B2AA0C

[22] Pierce RP Stevermer JJ. Disparities in use of telehealth at the onset of the COVID-19 public health emergency. J Telemed Telecare. (2020) 1357633X20963893. 10.1177/1357633X20963893 33081595 PMC7578842

[23] Weber E Miller SJ Astha V Janevic T Benn E. Characteristics of telehealth users in NYC for COVID-related care during the coronavirus pandemic. J Am Med Inform Assoc. (2020) 27:1949–54. 10.1093/jamia/ocaa216 32866249 PMC7499577

[24] Robles-Fradet A. Abortion Is Health Care. Available online at: https://healthlaw.org/resource/abortion-is-health-care/ (accessed September 9, 2021).

[25] Goodwin M. The Racist History of Abortion and Midwifery Bans. Available online at: https://www.aclu.org/news/racial-justice/the-racist-history-of-abortion-and-midwifery-bans/ (accessed September 9, 2021).

[26] Kaiser Family Foundation. The Availability Use of Medication Abortion [Internet]. San Francisco, CA: Kaiser Family Foundation (2021). Available online at: https://www.kff.org/womens-health-policy/fact-sheet/the-availability-and-use-of-medication-abortion/

[27] Food Drug Administration Approved Risk Evaluation Mitigation Strategies (REMS). Available online at: https://www.accessdata.fda.gov/scripts/cder/rems/index.cfm?event=RemsDetails.page&REMS=390 (accessed September 18, 2021).

[28] U.S. Food & Drug Administration. Questions and Answers on Mifeprex [Internet]. FDA (2021). Available online at: https://www.fda.gov/drugs/postmarket-drug-safety-information-patients-and-providers/questions-and-answers-mifeprex

[29] Fulcher IR Neill S Bharadwa S Goldberg AB Janiak E. State and federal abortion restrictions increase risk of COVID-19 exposure by mandating unnecessary clinic visits. Contraception. (2020) 102:385–91. 10.1016/j.contraception.2020.08.017 32905791 PMC7474961

[30] Jerman J Jones RK Onda T. Characteristics of U.S. Abortion Patients in 2014 and Changes Since 2008 (2016).

[31] Xue Y Smith JA Spetz J. Primary care nurse practitioners physicians in low-income rural areas, 2010-2016. JAM. (2019) 321 A:102–5. 10.1001/jama.2018.17944 30620363 PMC6583766

[32] Benkert R Hollie B Nordstrom CK Wickson B Bins-Emerick L. Trust mistrust, racial identity and patient satisfaction in Urban African American primary care patients of nurse practitioners. J Nurs Scholarsh. (2009) 41:211–9. 10.1111/j.1547-5069.2009.01273.x 19538706

[33] Byrd WM Clayton LA. Race, medicine, and health care in the United States: a historical survey. J Natl Med Assoc [Internet]. (2001) 93(3 Suppl):11S–34S. 12653395 PMC2593958

[34] Frey WH. Six Maps that Reveal America's Expanding Racial Diversity [Internet]. Brookings (2019). Available from: https://www.brookings.edu/research/americas-racial-diversity-in-six-maps/

[35] Planned Parenthood Map: Abortion Laws and Access in the South. Available online at: https://www.plannedparenthoodaction.org/issues/abortion/map-abortion-access-south (accessed September 9, 2021).

[36] Sobel L Ramaswarmy A Frederiksen B Salganicoff A. State Action to Limit Abortion Access During the COVID-19 Pandemic [Internet]. San Francisco, CA: Kaiser Family Foundation (2020). p. 1–3. Available online at: https://www.kff.org/coronavirus-covid-19/issue-brief/state-action-to-limit-abortion-access-during-the-covid-19-pandemic/

[37] Fuentes L Jerman J. Distance traveled to obtain clinical abortion care in the United States and reasons for clinic choice. J Womens Health. (2019) 28:1623–31. 10.1089/jwh.2018.7496 31282804 PMC6919239

[38] Centers for Medicare & Medicaid Services,. Trump Administration Drives Telehealth Services in Medicaid Medicare [Internet]. (2020). Available online at: https://www.cms.gov/newsroom/press-releases/trump-administration-drives-telehealth-services-medicaid-and-medicare

[39] State Medicaid & CHIP Telehealth Toolkit. Policy Considerations for States Expanding Use of Telehealth. COVID-19 Version [Internet]. Washington, DC: Centers for Medicare & Medicaid Services.; 2020. Available from: https://www.medicaid.gov/medicaid/benefits/downloads/medicaid-chip-telehealth-toolkit.pdf

[40] Casetext American College of Obstetricians & Gynecologists v. U.S. Food & Drug Admin., 472 F. Supp. 3d 183 |D. Md. (2020). Available online at: https://casetext.com/case/am-coll-of-obstetricians-gynecologists-v-us-food-drug-admin-1 (accessed September 9, 2021).

[41] Lindberg LD VandeVusse A Mueller J Kirstein M. Early Impacts of the COVID-19 Pandemic: Findings from the 2020 Guttmacher Survey of Reproductive Health Experiences. Guttmacher Institute (2020). p. 1–14. Available online at: https://www.guttmacher.org/report/early-impacts-covid-19-pandemic-findings-2020-guttmacher-survey-reproductive-health

[42] Congress. 123 Cong. Rec. (Bound) - December 7, 1977 [Internet]. govinfo.gov. U.S. Government Publishing Office (1977). Available online at: https://www.govinfo.gov/content/pkg/GPO-CRECB-1977-pt30/pdf/GPO-CRECB-1977-pt30-2.pdf

[43] Office of the Federal Register NA RA. Public Law 115 - 245 - Department of Defense and Labor, Health and Human Services, and Education Appropriations Act, 2019 and Continuing Appropriations Act, 2019 [Internet]. govinfo.gov. U.S. Government Publishing Office (2018). Available online at: https://www.govinfo.gov/app/details/PLAW-115publ245

[44] Guttmacher Institute Regulating Insurance Coverage of Abortion. Available online at: https://www.guttmacher.org/state-policy/explore/regulating-insurance-coverage-abortion (accessed September 9, 2021).

[45] Lynch EE Malcoe LH Laurent SE Richardson J Mitchell BC Meier HCS. The legacy of structural racism: associations between historic redlining, current mortgage lending, and health. SSM Popul Health. (2021) 14:100793. 10.1016/j.ssmph.2021.100793 33997243 PMC8099638

[46] The United States Department of Justice The Fair Housing Act. Available online at: https://www.justice.gov/crt/fair-housing-act-1 (accessed September 9, 2021).

[47] National Community Reinvestment Coalition. Redlining and Neighborhood Health [Internet]. Washington DC: National Community Reinvestment Coalition (2020). Available online at: https://ncrc.org/holc-health/

[48] CNET the Broadband Gap's Dirty Secret: Redlining Still Exists in Digital. Available online at: https://www.cnet.com/features/the-broadband-gaps-dirty-secret-redlining-still-exists-in-digital-form/ (accessed September 9 2021).

[49] Siefer A Callahan B. Limiting Broadband Investment to "Rural Only” Discriminates Against Black Americans and Other Communities of Color. Available online at: https://www.digitalinclusion.org/digital-divide-and-systemic-racism/ (accessed September 9, 2021).

[50] Sachs JW Graven P Gold JA Kassakian SZ. Disparities in telephone and video telehealth engagement during the COVID-19 pandemic. JAMIA Open. (2021) 4. 10.1093/jamiaopen/ooab056 34632322 PMC8496485

[51] Juckett G Unger K. Appropriate use of medical interpreters. AFP [Internet]. (2014) 90:476–80. Available online at: https://www.aafp.org/afp/2014/1001/p476.html 25369625

[52] Youdelman M. Summary of State Law Requirements Addressing Language Needs in Health Care. Available online at: https://healthlaw.org/resource/summary-of-state-law-requirements-addressing-language-needs-in-health-care-2/ (accessed September 9, 2021).

[53] Chandrashekar P. The Health Care System Is Shortchanging Non-English Speakers. Available online at: https://www.scientificamerican.com/article/the-health-care-system-is-shortchanging-non-english-speakers/ (accessed September 9, 2021).

[54] National Health Law Program. Discriminatory Provision of COVID-19 Services to Persons with Limited English Proficiency [Internet]. Washington, DC (2021). p. 19. Available online at: https://healthlaw.org/wp-content/uploads/2021/04/OCR-LEP-Complaint-4-30-21-for-publication.pdf

[55] Shah SA Velasquez DE Song Z. Reconsidering reimbursement for medical interpreters in the era of COVID-19. JAMA Health Forum. (2020) 1(10):e201240. 10.1001/jamahealthforum.2020.1240 36218550

[56] California Pan-Ethnic Health Network. Equity in the Age of Telehealth: Considerations for California Policymakers [Internet]. Sacramento, CA: California Pan-Ethnic Health Network (2020). p. 6–11. Available online at: https://cpehn.org/assets/uploads/2020/12/telehealthfactsheet-12420-d-1.pdf

[57] Aiken A Lohr PA Lord J Ghosh N Starling J. Effectiveness, safety and acceptability of no-test medical abortion (Termination of Pregnancy) provided via telemedicine: a national cohort study. BJOG. (2021). 10.22541/au.160769559.97160234/v1 33605016 PMC8360126

[58] Chong E Shochet T Raymond E Platais I Anger HA Raidoo S. Expansion of a direct-to-patient telemedicine abortion service in the United States and experience during the COVID-19 pandemic. Contraception. (2021) 104:43–8. 10.1016/j.contraception.2021.03.019 33781762 PMC9748604

[59] Duckworth TS. 1021 - 117th Congress (2021-2022): EACH Act of 2021. Available online at: https://www.congress.gov/bill/117th-congress/senate-bill/1021/text (accessed September 9, 2021).

[60] The Infrastructure Bill Is the Biggest Thing Congress Ever Did for Broadband [Internet]. Free Press. [cited 2022 Jan 20]. Available from: https://www.freepress.net/our-response/expert-analysis/explainers/infrastructure-bill-biggest-thing-congress-ever-did

[61] Pew Research Center,. Demographics of Mobile Device Ownership Adoption in the United States [Internet]. Washington, DC: Pew Research Center (2021). p. 1–7. Available online at: https://www.pewresearch.org/internet/fact-sheet/mobile/

[62] Ray R Sewell AA Gilbert KL Roberts JD. Missed opportunity? Leveraging mobile technology to reduce racial health disparities. J Health Polit Policy Law. (2017) 42:901–24. 10.1215/03616878-3940477 28663182

